# Reduced reverse degree-based topological indices of graphyne and graphdiyne nanoribbons with applications in chemical analysis

**DOI:** 10.1038/s41598-023-51112-1

**Published:** 2024-01-04

**Authors:** Shahid Zaman, K. H. Hakami, Sadaf Rasheed, Fekadu Tesgera Agama

**Affiliations:** 1 Department of Mathematics, University of Sialkot, Sialkot, 51310 Pakistan; 2 Department of Mathematics, Faculty of Science, Jazan University, 45142 Jazan, Saudi Arabia; 3 Department of Mathematics, Wollega University, 395 Nekemte, Ethiopia

**Keywords:** Applied mathematics, Computational science

## Abstract

Graphyne and Graphdiyne Nanoribbons reveal significant prospective with diverse applications. In electronics, they propose unique electronic properties for high-performance nanoscale devices, while in catalysis, their excellent surface area and reactivity sort them valuable catalyst supports for numerous chemical reactions, contributing to progresses in sustainable energy and environmental remediation. The topological indices (TIs) are numerical invariants that provide important information about the molecular topology of a given molecular graph. These indices are essential in QSAR/QSPR analysis and play a significant role in predicting various physico-chemical characteristics. In this article, we present a formula for computing reduced reverse (RR) degree-based topological indices for graphyne and graphdiyne nanoribbons, including the RR Zagreb indices, RR hyper-Zagreb indices, RR forgotten index, RR atom bond connectivity index, and RR Geometric-arithmetic index. We also execute a graph-theoretical analysis and comparison to demonstrate the critical significance and validate the acquired results. Our findings provide insights into the structural and chemical properties of these nanoribbons and contribute to the development of new materials for various applications.

## Introduction

Graphene derivatives such as graphyne and graphdiyne have attracted significant attention in various fields of mathematics and science due to their unique electronic, optical, and mechanical properties. Some of the mathematical applications of these materials are as follows:

In combinatorics, Graphyne and graphdiyne represented as molecular graphs, which have combinatorial properties that can be analyzed using graph theory. The study of these materials involves graph-theoretical concepts such as graph isomorphism, graph coloring, and graph embedding. Since, the numerical invariants known as topological indices that provide information about the topology of a molecular graph in this way Graphyne and graphdiyne used to study the behavior of various topological indices, such as the Zagreb indices, forgotten index, atom bond connectivity index, and geometric-arithmetic index.

In quantum mechanics the graphene derivatives are useful to study the behavior of electrons in a two-dimensional lattice structure. The electronic properties of these materials can be analyzed using quantum mechanics, methods such as density functional theory (DFT) and the tight-binding approximation (TBA) have been employed to predict the electronic structure of these materials.

Overall, the mathematical applications of graphene derivatives such as graphyne and graphdiyne are diverse and span multiple fields, making these materials important for future research and development. The physical properties of both structures are given by the corresponding determined topological indices^1–5^.

The compressive energy of metallic element has been discovered to be greatly enhanced and to outweigh the addition of metal elements and metallic binding to these carbonaceous materials. These materials have nearly uniform metallic atom charges that enable them to bind molecules with much lower sorption energies^6^. The calculated maximum sorption enthalpy for near-room-temperature element storage (3.6 kcal/mol) closely matches the key computed enthalpies for element sorption (ranging from 3.5 to 2.8 kcal/mol). These planar carbon allotropes have potential applications in microelectronics and tunable bandgaps achieved by adjusting the number of aliphatically linked bridging units.

Assuming $G$ is a graph, the definitions and notations used, such as $d(u)$ to denote the degree of vertex u, are derived from the referenced book^7^. Graph invariants might be utilized to evaluate the graphical structures of chemical substances using topological indices (TIs). By converting a chemical graph to a numerical number, TIs are essentially represented. Wiener makes the suggestion to use Tis in 1947. Initially, he described this index ($W$) on tress and discussed how it was used to relate the physical characteristics of alcohols, alkanes, and related complexes^8^.

Cheminformatics is a developing discipline that assists (QSARs) and (QSPRs) are commonly used to predict the bioactivities and possessions of chemical compounds^9–11^. The bioactivity of organic compounds has been predicted using topological indices and physico-chemical properties^12–15^.

In a chemical graph, the vertices stand in for atoms or compounds, though the contacts represent their chemical interactions. Topological indices, which describe the structure of the graph, and numerical graph invariants. According to^16^, the degree in any vertex is represented with ${d}_{u}$ or $d(u)$ and represent the number of edges that intersect that vertex $u$. Subsequently it is extra cost-effective method of testing compounds than testing them in a wet lab, numerous researchers are currently conducting QSPR analyses of different molecules^11,17–22^.

The Computation of degree-based topological indices for porphyrazine and tetrakis porphyrazine are studied in^23^. On topological properties of boron triangular sheet are characterized in^24^. Some other degree based topological indices are discussed in^25–30^. The use of carbon micro-tubes, general bridge graphs, plus chemical graph by products is studied by^31,32^. The topological characteristics of mental-organic structures were covered by^33^. In^34^ the estimated degree based TD for hexagon star network. In addition, QSPR examination may be used to create models that predict the characteristics or functions of organic chemical compounds.

In this article, we have presented results for calculating reduced reverse degree-based topological indices (TIs) for graphyne and graphdiyne. Notably, our work builds upon the foundation laid by^35^ developed a method for analyzing TI. It is worth highlighting that our study marks a pioneering effort, as we are the first to calculate TIs for nanostructures, a groundbreaking achievement documented by^36^. Subsequent to this milestone, researchers^37^ have also extended these calculations to include nanotubes.

## Preliminaries

In 1736, Leonhard Euler laid the foundation of graph theory and foreshadowed the idea of topology. In 1947, Wiener introduced the concept of topological indices, considering both their practical applications and theoretical significance^38^.

The Wiener index of a graph G is defined as:

$$W\left(G\right)=\frac{1}{2}\sum_{( u , v)\in V(G)}d(u,v)$$

where $(u,v)$ show the order pair of vertices.

Gutman and coauthor introduced the Zagreb index denoted by ${M}_{1}(G)$. It is very important topological index as defined in^39^.

$${M}_{1}\left(G\right)=\sum_{uv\in E(G)}({d}_{u}+{d}_{v}).$$

Similarly, the second Zagreb index of a graph G are mathematically represented as:

$${M}_{2} \left(G\right) = {\sum }_{uv\in E (G)}({d}_{u}{d}_{{\text{v}}}).$$

In ^40^ the authors Kulli, presented the concept of reverse vertex degree $R(v)$, demarcated as:

$$R\left(v\right)=\Delta \left(G\right)-d\left(v\right)+1.$$

Encouraged by this definition, ^41^ defined the reduced reverse degree as:

$$RR\left(v\right)=\Delta \left(G\right)-d\left(v\right)+2.$$

It was developed to investigate the influence of low reverse degree in QSPR analysis. They also established the Zagreb index, F-index, ABC index, and we analyzed the relationship between physico-chemical characteristics of various COVID-19 drugs and a simplified reverse degree-based version of arithmetic index.

The reduced reverse Zagreb indices^39,42,43^, which is defined as:

$$ \begin{aligned} RRM_{1} \left( G \right) & = \mathop \sum \limits_{uv \in E} \left[ {RR\left( u \right) + RR\left( v \right)} \right] \\ RRM_{2} \left( G \right) & = \mathop \sum \limits_{uv \in E} \left[ {RR\left( u \right)*RR\left( v \right)} \right] \\ \end{aligned} $$

The reduced reverse hyper-Zagreb indices ^41^, that is denoted as:

$$ \begin{aligned} RRHM_{1} \left( G \right) & = \mathop \sum \limits_{uv \in E} \left[ {RR\left( u \right) + RR\left( v \right)} \right]^{2} \\ RRHM_{2} \left( G \right) & = \mathop \sum \limits_{uv \in E} \left[ {RR\left( u \right)*RR\left( v \right)} \right]^{2} \\ \end{aligned} $$

Furtula and Gutman^19^ is defined the reduced reverse forgotten index as:

$$RRF\left(G\right)=\sum_{uv\in E}[RR{(u)}^{2}+RR{(v)}^{2}]$$

The reduced reverse atom bond connectivity index ^44^, described as:

$$RRABC\left(G\right)=\sum_{uv\in E}\left[\frac{RR\left(u\right)+RR\left(v\right)-2}{RR\left(u\right)*RR(v)}\right]$$

And, the reduced reverse Geometric-arithmetic index ^45^, is represented as:

$$RRGA\left(G\right)=\sum_{uv\in E}\left[\frac{2\sqrt{RR\left(u\right)*RR(v)}}{RR\left(u\right)+RR(v)}\right]$$

## Molecular structures of graphyne and graphdiyne:

**Figure 1:**
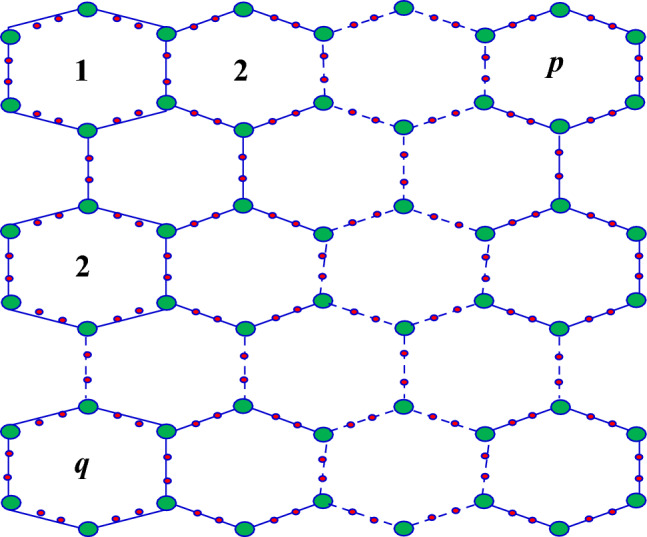
Structure of a $$\alpha -graphyne(\alpha -Gy)$$.

**Figure 2:**
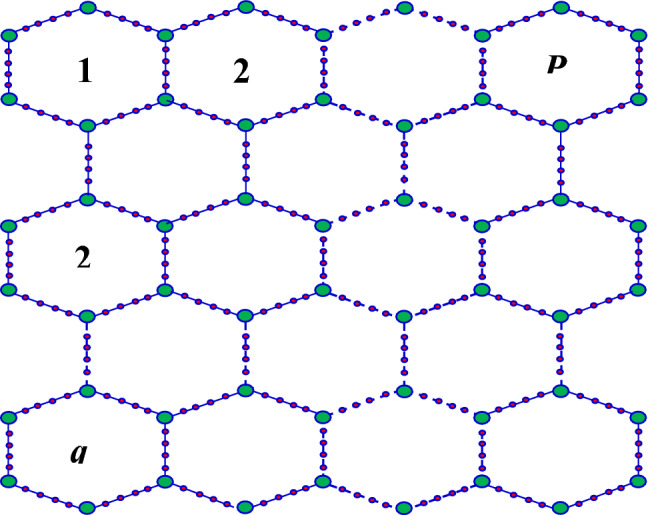
Structure of a $$\alpha -graphdiyne (\alpha -Gd)$$.

–C≡C– is inserted among each C–C bond into the 2D hexagonal network of graphene is stationary in nature, which makes it a distinct class $\alpha$-graphene. Thus, the prediction of $\alpha$-graphene has opened the door to the synthesis of $\alpha$-graphene, in graphdiyne, each carbon–carbon bond in the graphene lattice is replaced by a diacetylene bond. Figures 1 and 2 show the conformation of $\alpha$-graphyne and $\alpha$-graphdiyne of measurement $\alpha -Gy$ and $\alpha -Gd$ respectively. In spite of the fact that graphdyne belong to the group Graphene, it attitudes out as gradually remarkable in the light of its appealing possessions. Arockiaraj et al.^46^ computed the quality-weighted factors for $\alpha -Gy$ and $\alpha -Gd$ since these two grids represent subdivisions of graphene with separately bond divided by 2 and 4 particles, respectively.

The fundamental data obligatory for studying chemical graphs are the atoms and their connections, which we utilized to develop our model. Additionally, we determined the number of bonds among carbon, hydrogen, and nitrogen atoms. As a conclusion, we have built the unitary structure of graphyne and graphdiyne. In 2022, the authors of^47^ demonstrated a formula for calculating any degree-based topological index for graphene and graphdiyne nanoribbons, motivated by their work we have computing reduced reverse (RR) degree-based topological indices for graphyne and graphdiyne nanoribbons. The structures of graphyne and graphdiyne are taken from^47^.

## Reduced reverse degree-based topological descriptors for the graphyne and graphdiyne

In this study, we computed the topological descriptors of graphyne and graphdiyne structures. In Tables 1 and 2 show the vertex partitons of the graphyne and graphdiyne structures, respectively, according to the degree base and reduced reverse degrees of the end vertices. For the sake of simplicity, we assume that u is any vertex of a graph G, $d\left(u\right)$ (resp. $\mathcal{R}\mathcal{R}\left(u\right)$) is a degree of u (resp. reduced reverse degree of u) and frequency reflects the number of vertices of same degree that appears in a given graph.

**Table 1:** Vertex partition of graphyne $(\alpha -Gy$).

$d\left(u\right)$	$\mathcal{R}\mathcal{R}\left(u\right)$	Frequency
2	3	$(12pq+6q)$
3	2	$(4pq-2p-2q)$

**Table 2:** Vertex partition of graphdiyne $(\alpha -Gd$).

$d\left(u\right)$	$\mathcal{R}\mathcal{R}\left(u\right)$	Frequency
2	3	$(24pq-2p+8q)$
3	2	$(4pq-2p-2q)$

The graphyne and graphdiyne edge partition based on the endpoint degrees of each edge are displayed in Tables 3 and 4. The maximum vertex degree of graphyne and graphdiyne is 3. By using the definition of reduced reverse vertex degree $RR\left(v\right)=\Delta \left(G\right)-d\left(v\right)+2$.

**Table 3:** Edge partition of graphyne based on degrees of end vertices of each edge.

$(d\left(u\right),d(v))$	Frequency
$(\mathrm{2,2})$	$(6pq+3p+9q)$
$(\mathrm{2,3})$	$(12pq-6p-6q)$

**Table 4:** Reduced reverse degree-based edge partition of graphyne $(\alpha -Gy$).

$(\mathcal{R}\mathcal{R}\left(u\right),\mathcal{R}\mathcal{R}(v))$	Frequency
$(\mathrm{3,3})$	$(6pq+3p+9q)$
$(\mathrm{3,2})$	$(12pq-6p-6q)$

Tables 5 and 6 shows the reduced reverse degree-based edge partition of graphyne and graphdiyne structures.

**Table 5:** Edge partition of graphdiyne based on degrees of end vertices of each edge.

$(d\left(u\right),d(v))$	Frequency
$(\mathrm{2,2})$	$(18pq+p+11q)$
$(\mathrm{2,3})$	$(12pq-6p-6q)$

**Table 6:** Reduced reverse degree-based edge partition of graphdiyne $(\alpha -Gd$).

$(\mathcal{R}\mathcal{R}\left(u\right),\mathcal{R}\mathcal{R}(v))$	Frequency
$(\mathrm{3,3})$	$(18pq+p+11q)$
$(\mathrm{3,2})$	$(12pq-6p-6q)$

Now, we describe a formula for determining reduced reverse degree-based topological indices for $\alpha -graphyne(\alpha -Gy)$:

$$ \begin{aligned} TI(\alpha - Gy) & = \mathop \sum \limits_{{uv \in E\left( {\alpha - Gy} \right)}} \left[ {RR\left( u \right),RR\left( v \right)} \right] \\ & = \mathop \sum \limits_{{uv \in E_{3, 3} }} RR\left( {3,3} \right) + \mathop \sum \limits_{{uv \in E_{3,2} }} RR\left( {3,2} \right) \\ & = \left( {6pq + 3p + 9q} \right)RR\left( {3,3} \right) + \left( {12pq - 6p - 6q} \right)RR\left( {3,2} \right) \\ TI(\alpha - Gy) & = 6pq\left[ {RR\left( {3,3} \right) + 2RR\left( {3,2} \right)} \right] + 3p\left[ {RR\left( {3,3} \right) - 2RR\left( {3,2} \right)} \right] \\ & \quad + 3q\left[ {3RR\left( {3,3} \right) - 2RR\left( {3,2} \right)} \right] \\ \end{aligned} $$

Now, we estimated the reduced reverse degree-based topological indices of $\alpha -graphyne(\alpha -Gy)$.

### Reduced reverse 1st Zagreb index for $graphyne (\alpha -Gy)$

$$ \begin{aligned} RRM_{1} (G) & = \mathop \sum \limits_{uv \in E} \left[ {RR(u) + RR(v)} \right] \\ & = 6pq\left[ {RR\left( {3,3} \right) + 2RR\left( {3,2} \right)} \right] + 3p\left[ {RR\left( {3,3} \right) - 2RR\left( {3,2} \right)} \right] \\ & \quad + 3q\left[ {3RR\left( {3,3} \right) - 2RR\left( {3,2} \right)} \right] \\ & = 6pq\left[ {\left( {3 + 3} \right) + 2\left( {3 + 2} \right)} \right] + 3p\left[ {\left( {3 + 3} \right) - 2\left( {3 + 2} \right)} \right] \\ & \quad + 3q\left[ {3\left( {3 + 3} \right) - 2\left( {3 + 2} \right)} \right] \\ & = 96pq - 12p + 24q \\ \end{aligned} $$

### Reduced reverse 2nd Zagreb index for $graphyne (\alpha -Gy)$

$$ \begin{aligned} RRM_{2} (G) & = \mathop \sum \limits_{uv \in E} \left[ {RR\left( u \right)*RR\left( v \right)} \right] \\ & = 6pq\left[ {RR\left( {3,3} \right) + 2RR\left( {3,2} \right)} \right] + 3p\left[ {RR\left( {3,3} \right) - 2RR\left( {3,2} \right)} \right] \\ & \quad + 3q\left[ {3RR\left( {3,3} \right) - 2RR\left( {3,2} \right)} \right] \\ & = 6pq\left[ {\left( {3*3} \right) + 2\left( {3*2} \right)} \right] + 3p\left[ {\left( {3*3} \right) - 2\left( {3*2} \right)} \right] \\ & \quad + 3q\left[ {3\left( {3*3} \right) - 2\left( {3*2} \right)} \right] \\ & = 126pq - 9p + 45q \\ \end{aligned} $$

### Reduced reverse 1st hyper-Zagreb index for $graphyne (\alpha -Gy)$

$$ \begin{aligned} RRHM_{1} (G) & = \mathop \sum \limits_{uv \in E} \left[ {RR\left( u \right) + RR\left( v \right)} \right]^{2} \\ & = 6pq\left[ {RR\left( {3,3} \right) + 2RR\left( {3,2} \right)} \right] + 3p\left[ {RR\left( {3,3} \right) - 2RR\left( {3,2} \right)} \right] \\ & \quad + 3q\left[ {3RR\left( {3,3} \right) - 2RR\left( {3,2} \right)} \right] \\ & = 6pq\left[ {\left( {3 + 3} \right)^{2} + 2\left( {3 + 2} \right)^{2} } \right] + 3p\left[ {\left( {3 + 3} \right)^{2} - 2\left( {3 + 2} \right)^{2} } \right] \\ & \quad + 3q\left[ {3\left( {3 + 3} \right)^{2} - 2\left( {3 + 2} \right)^{2} } \right] \\ & = 516pq - 42p + 174q \\ \end{aligned} $$

### Reduced reverse 2nd hyper-Zagreb index for $graphyne (\alpha -Gy)$

$$ \begin{aligned} RRHM_{2} (G) & = \mathop \sum \limits_{uv \in E} \left[ {RR\left( u \right)*RR\left( v \right)} \right]^{2} \\ & = 6pq\left[ {RR\left( {3,3} \right) + 2RR\left( {3,2} \right)} \right] + 3p\left[ {RR\left( {3,3} \right) - 2RR\left( {3,2} \right)} \right] \\ & \quad + 3q\left[ {3RR\left( {3,3} \right) - 2RR\left( {3,2} \right)} \right] \\ & = 6pq\left[ {\left( {3*3} \right)^{2} + 2\left( {3*2} \right)^{2} } \right] + 3p\left[ {\left( {3*3} \right)^{2} - 2\left( {3*2} \right)^{2} } \right] \\ & \quad + 3q\left[ {3\left( {3*3} \right)^{2} - 2\left( {3*2} \right)^{2} } \right] \\ & = 918pq + 27p + 513q \\ \end{aligned} $$

### Reduced reverse forgotten index for $graphyne (\alpha -Gy)$

$$ \begin{aligned} RRF(G) & = \mathop \sum \limits_{uv \in E} \left[ {RR\left( u \right)^{2} + RR\left( v \right)^{2} } \right] \\ & = 6pq\left[ {RR\left( {3,3} \right) + 2RR\left( {3,2} \right)} \right] + 3p\left[ {RR\left( {3,3} \right) - 2RR\left( {3,2} \right)} \right] \\ & \quad + 3q\left[ {3RR\left( {3,3} \right) - 2RR\left( {3,2} \right)} \right] \\ & = 6pq\left[ {\left( {3^{2} + 3^{2} } \right) + 2\left( {3^{2} + 2^{2} } \right)} \right] + 3p\left[ {\left( {3^{2} + 3^{2} } \right) - 2\left( {3^{2} + 2^{2} } \right)} \right] \\ & \quad + 3q\left[ {3\left( {3^{2} + 3^{2} } \right) - 2\left( {3^{2} + 2^{2} } \right)} \right] \\ & = 264pq - 24p + 84q \\ \end{aligned} $$

### Reduced reverse atom bond connectivity (ABC) index for $graphyne (\alpha -Gy)$

$$ \begin{aligned} RRABC(G) & = \mathop \sum \limits_{uv \in E} \left[ {\frac{RR\left( u \right) + RR\left( v \right) - 2}{{RR\left( u \right)*RR\left( v \right)}}} \right] \\ & = 6pq\left[ {RR\left( {3,3} \right) + 2RR\left( {3,2} \right)} \right] + 3p\left[ {RR\left( {3,3} \right) - 2RR\left( {3,2} \right)} \right] \\ & \quad + 3q\left[ {3RR\left( {3,3} \right) - 2RR\left( {3,2} \right)} \right] \\ & = 6pq\left[ {\left( {\frac{3 + 3 - 2}{{3*3}}} \right) + 2\left( {\frac{3 + 2 - 2}{{3*2}}} \right)} \right] + 3p\left[ {\left( {\frac{3 + 3 - 2}{{3*3}}} \right) - 2\left( {\frac{3 + 2 - 2}{{3*2}}} \right)} \right] \\ & \quad + 3q\left[ {3\left( {\frac{3 + 3 - 2}{{3*3}}} \right) - 2\left( {\frac{3 + 2 - 2}{{3*2}}} \right)} \right] \\ & = \frac{26}{3}pq - \frac{5}{3}p + q \\ \end{aligned} $$

### Reduced reverse geometric-arithmetic index for $graphyne (\alpha -Gy)$

$$ \begin{aligned} RRGA(G) & = \mathop \sum \limits_{uv \in E} \left[ {\frac{{2\sqrt {RR\left( u \right)*RR\left( v \right)} }}{RR\left( u \right) + RR\left( v \right)}} \right] \\ & = 6pq\left[ {RR\left( {3,3} \right) + 2RR\left( {3,2} \right)} \right] + 3p\left[ {RR\left( {3,3} \right) - 2RR\left( {3,2} \right)} \right] \\ & \quad + 3q\left[ {3RR\left( {3,3} \right) - 2RR\left( {3,2} \right)} \right] \\ & = 6pq\left[ {\left( {\frac{{2\sqrt {3*3} }}{3 + 3}} \right) + 2\left( {\frac{{2\sqrt {3*2} }}{3 + 2}} \right)} \right] + 3p\left[ {\left( {\frac{{2\sqrt {3*3} }}{3 + 3}} \right) - 2\left( {\frac{{2\sqrt {3*2} }}{3 + 2}} \right)} \right] \\ & \quad + 3q\left[ {3\left( {\frac{{2\sqrt {3*3} }}{3 + 3}} \right) - 2\left( {\frac{{2\sqrt {3*2} }}{3 + 2}} \right)} \right] \\ & = 6pq\left( {\frac{5 + 4\sqrt 6 }{5}} \right) + 3p\left( {\frac{5 - 4\sqrt 6 }{5}} \right) + 3q\left( {\frac{15 - 4\sqrt 6 }{5}} \right) \\ \end{aligned} $$

Now, we introduce a method to compute reduced reverse degree-based topological indices for $\alpha -graphdiyne(\alpha -Gd)$:

$$ \begin{aligned} TI\left( {\alpha - Gd} \right) & = \mathop \sum \limits_{{uv \in E\left( {\alpha - Gd} \right)}} \left[ {RR\left( u \right),RR\left( v \right)} \right] \\ & = \mathop \sum \limits_{{uv \in E_{3, 3} }} RR\left( {3,3} \right) + \mathop \sum \limits_{{uv \in E_{3,2} }} RR\left( {3,2} \right) \\ & = \left( {18pq + p + 11q} \right)RR\left( {3,3} \right) + \left( {12pq - 6p - 6q} \right)RR\left( {3,2} \right) \\ TI\left( {\alpha - Gy} \right) & = 6pq\left[ {3RR\left( {3,3} \right) + 2RR\left( {3,2} \right)} \right] + p\left[ {RR\left( {3,3} \right) - 6RR\left( {3,2} \right)} \right] \\ & \quad + q\left[ {11RR\left( {3,3} \right) - 6RR\left( {3,2} \right)} \right] \\ \end{aligned} $$

Also, we estimated the reduced reverse degree-based topological indices of $\alpha -graphdiyne(\alpha -Gd)$.

### Reduced reverse 1st Zagreb index for $graphdiyne (\alpha -Gd)$

$$ \begin{aligned} RRM_{1} (G) & = \mathop \sum \limits_{uv \in E} \left[ {RR\left( u \right) + RR\left( v \right)} \right] \\ & = 6pq\left[ {3RR\left( {3,3} \right) + 2RR\left( {3,2} \right)} \right] + p\left[ {RR\left( {3,3} \right) - 6RR\left( {3,2} \right)} \right] \\ & \quad + q\left[ {11RR\left( {3,3} \right) - 6RR\left( {3,2} \right)} \right] \\ & = 6pq\left[ {3\left( {3 + 3} \right) + 2\left( {3 + 2} \right)} \right] + p\left[ {\left( {3 + 3} \right) - 6\left( {3 + 2} \right)} \right] \\ & \quad + q\left[ {11\left( {3 + 3} \right) - 6\left( {3 + 2} \right)} \right] \\ & = 168pq - 24p + 36q \\ \end{aligned} $$

$$ = 168pq - 24p + 36q $$

### Reduced reverse 2nd Zagreb index for $graphdiyne (\alpha -Gd)$

$$ \begin{aligned} RRM_{2} \left( G \right) & = \mathop \sum \limits_{uv \in E} \left[ {RR\left( u \right)*RR\left( v \right)} \right] \\ & = 6pq\left[ {3RR\left( {3,3} \right) + 2RR\left( {3,2} \right)} \right] + p\left[ {RR\left( {3,3} \right) - 6RR\left( {3,2} \right)} \right] \\ & \quad + q\left[ {11RR\left( {3,3} \right) - 6RR\left( {3,2} \right)} \right] \\ & = 6pq\left[ {3\left( {3*3} \right) + 2\left( {3*2} \right)} \right] + p\left[ {\left( {3*3} \right) - 6\left( {3*2} \right)} \right] \\ & \quad + q\left[ {11\left( {3*3} \right) - 6\left( {3*2} \right)} \right] \\ & = 234pq - 27p + 63q \\ \end{aligned} $$

### Reduced reverse 1st hyper-Zagreb index for $graphdiyne (\alpha -Gd)$

$$ \begin{aligned} RRHM_{1} (G) & = \mathop \sum \limits_{uv \in E} \left[ {RR\left( u \right) + RR\left( v \right)} \right]^{2} \\ & = 6pq\left[ {3RR\left( {3,3} \right) + 2RR\left( {3,2} \right)} \right] + p\left[ {RR\left( {3,3} \right) - 6RR\left( {3,2} \right)} \right] \\ & \quad + q\left[ {11RR\left( {3,3} \right) - 6RR\left( {3,2} \right)} \right] \\ & = 6pq\left[ {3\left( {3 + 3} \right)^{2} + 2\left( {3 + 2} \right)^{2} } \right] + p\left[ {\left( {3 + 3} \right)^{2} - 6\left( {3 + 2} \right)^{2} } \right] \\ & \quad + q\left[ {11\left( {3 + 3} \right)^{2} - 6\left( {3 + 2} \right)^{2} } \right] \\ & = 948pq - 114p + 246q \\ \end{aligned} $$

### Reduced reverse 2nd hyper-Zagreb index for $graphdiyne (\alpha -Gd)$

$$ \begin{aligned} RRHM_{2} (G) & = \mathop \sum \limits_{uv \in E} \left[ {RR\left( u \right)*RR\left( v \right)} \right]^{2} \\ & = 6pq\left[ {3RR\left( {3,3} \right) + 2RR\left( {3,2} \right)} \right] + p\left[ {RR\left( {3,3} \right) - 6RR\left( {3,2} \right)} \right] \\ & \quad + q\left[ {11RR\left( {3,3} \right) - 6RR\left( {3,2} \right)} \right] \\ & = 6pq\left[ {3\left( {3*3} \right)^{2} + 2\left( {3*2} \right)^{2} } \right] + p\left[ {\left( {3*3} \right)^{2} - 6\left( {3*2} \right)^{2} } \right] \\ & \quad + q\left[ {11\left( {3*3} \right)^{2} - 6\left( {3*2} \right)^{2} } \right] \\ & = 1890pq - 135p + 675q \\ \end{aligned} $$

### Reduced reverse forgotten index for $graphdiyne (\alpha -Gd)$

$$ \begin{aligned} RRF\left( G \right) & = \mathop \sum \limits_{uv \in E} \left[ {RR\left( u \right)^{2} + RR\left( v \right)^{2} } \right] \\ & = 6pq\left[ {3RR\left( {3,3} \right) + 2RR\left( {3,2} \right)} \right] + p\left[ {RR\left( {3,3} \right) - 6RR\left( {3,2} \right)} \right] \\ & \quad + q\left[ {11RR\left( {3,3} \right) - 6RR\left( {3,2} \right)} \right] \\ & = 6pq\left[ {3\left( {3^{2} + 3^{2} } \right) + 2\left( {3^{2} + 2^{2} } \right)} \right] + p\left[ {\left( {3^{2} + 3^{2} } \right) - 6\left( {3^{2} + 2^{2} } \right)} \right] \\ & \quad + q\left[ {11\left( {3^{2} + 3^{2} } \right) - 6\left( {3^{2} + 2^{2} } \right)} \right] \\ & = 480pq - 60p + 120q \\ \end{aligned} $$

### Reduced reverse atom bond connectivity (ABC) index for $graphdiyne (\alpha -Gd)$

$$ \begin{aligned} RRABC\left( G \right) & = \mathop \sum \limits_{uv \in E} \left[ {\frac{RR\left( u \right) + RR\left( v \right) - 2}{{RR\left( u \right)*RR\left( v \right)}}} \right] \\ & = 6pq\left[ {3RR\left( {3,3} \right) + 2RR\left( {3,2} \right)} \right] + p\left[ {RR\left( {3,3} \right) - 6RR\left( {3,2} \right)} \right] \\ & \quad + q\left[ {11RR\left( {3,3} \right) - 6RR\left( {3,2} \right)} \right] \\ & = 6pq\left[ {3\left( {\frac{3 + 3 - 2}{{3*3}}} \right) + 2\left( {\frac{3 + 2 - 2}{{3*2}}} \right)} \right] + p\left[ {\left( {\frac{3 + 3 - 2}{{3*3}}} \right) - 6\left( {\frac{3 + 2 - 2}{{3*2}}} \right)} \right] \\ & \quad + q\left[ {11\left( {\frac{3 + 3 - 2}{{3*3}}} \right) - 6\left( {\frac{3 + 2 - 2}{{3*2}}} \right)} \right] \\ & = 14pq - \frac{23}{9}p + \frac{17}{9}q \\ \end{aligned} $$

### Reduced reverse Geometric-arithmetic index for $graphdiyne (\alpha -Gd)$

$$ \begin{aligned} RRGA(G) & = \mathop \sum \limits_{uv \in E} \left[ {\frac{{2\sqrt {RR\left( u \right)*RR\left( v \right)} }}{RR\left( u \right) + RR\left( v \right)}} \right] \\ & = 6pq\left[ {3RR\left( {3,3} \right) + 2RR\left( {3,2} \right)} \right] + p\left[ {RR\left( {3,3} \right) - 6RR\left( {3,2} \right)} \right] \\ & \quad + q\left[ {11RR\left( {3,3} \right) - 6RR\left( {3,2} \right)} \right] \\ & = 6pq\left[ {3\left( {\frac{{2\sqrt {3*3} }}{3 + 3}} \right) + 2\left( {\frac{{2\sqrt {3*2} }}{3 + 2}} \right)} \right] + p\left[ {\left( {\frac{{2\sqrt {3*3} }}{3 + 3}} \right) - 6\left( {\frac{{2\sqrt {3*2} }}{3 + 2}} \right)} \right] \\ & \quad + q\left[ {11\left( {\frac{{2\sqrt {3*3} }}{3 + 3}} \right) - 6\left( {\frac{{2\sqrt {3*2} }}{3 + 2}} \right)} \right] \\ & = 6pq\left( {\frac{15 + 4\sqrt 6 }{5}} \right) + p\left( {\frac{5 - 12\sqrt 6 }{5}} \right) + q\left( {\frac{55 - 12\sqrt 6 }{5}} \right) \\ \end{aligned} $$

## Numerical and graphical discussion for computed results

For graphyne and grapdiyne, we have calculated the topological indices, which depends on the sharp weight. The closed formulas for reduced reverse degree based TI’s, are provided. The comparison of these topological indices for graphyne and graphdiyne nanoribbons provide insights into their structural and chemical characteristics. In Tables 7 and 8, all the TI’s reflects the connectivity of atoms for the considered graphs. The more value of the TI shows the strong connectivity and the less value shows the weak connectivity. For all considered topological indices the value of $RRH{M}_{2}\left(G\right)$ is higher than the other topological indices. This shows that $RRH{M}_{2}\left(G\right)$ gives more connectivity for both graphs than other topological indices as shown in Tables 7 and 8.

**Table 7:** Numerical behavior of topological indices for Graphyne $(\alpha -Gy)$.

N	$RR{M}_{1}\left(G\right)$	$RR{M}_{2}\left(G\right)$	$RRH{M}_{1}\left(G\right)$	$RRH{M}_{2}\left(G\right)$	$RRF\left(G\right)$	$RRABC\left(G\right)$	$RRGA\left(G\right)$
1	108	162	648	1458	324	8	18
2	408	576	2328	4752	1176	33.33	71.53
3	900	1242	5040	9882	2556	76	160.55
4	1584	2160	8784	16,848	4464	136	285.09
5	2460	3330	13,560	25,650	6900	213.33	445.15

**Table 8:** Numerical behavior of topological indices for Graphdiyne $(\alpha -Gd)$.

N	$RR{M}_{1}\left(G\right)$	$RR{M}_{2}\left(G\right)$	$RRH{M}_{1}\left(G\right)$	$RRH{M}_{2}\left(G\right)$	$RRF\left(G\right)$	$RRABC\left(G\right)$	$RRGA\left(G\right)$
1	180	270	1080	2430	540	13.33	30
2	696	1008	4056	8640	2040	54.67	119.51
3	1548	2214	8928	18,630	4500	124	268.54
4	2736	3888	15,696	32,400	7920	221.33	477.09
5	4260	6030	24,360	49,950	12,300	346.67	745.15

## Conclusion

**Figure 3:**
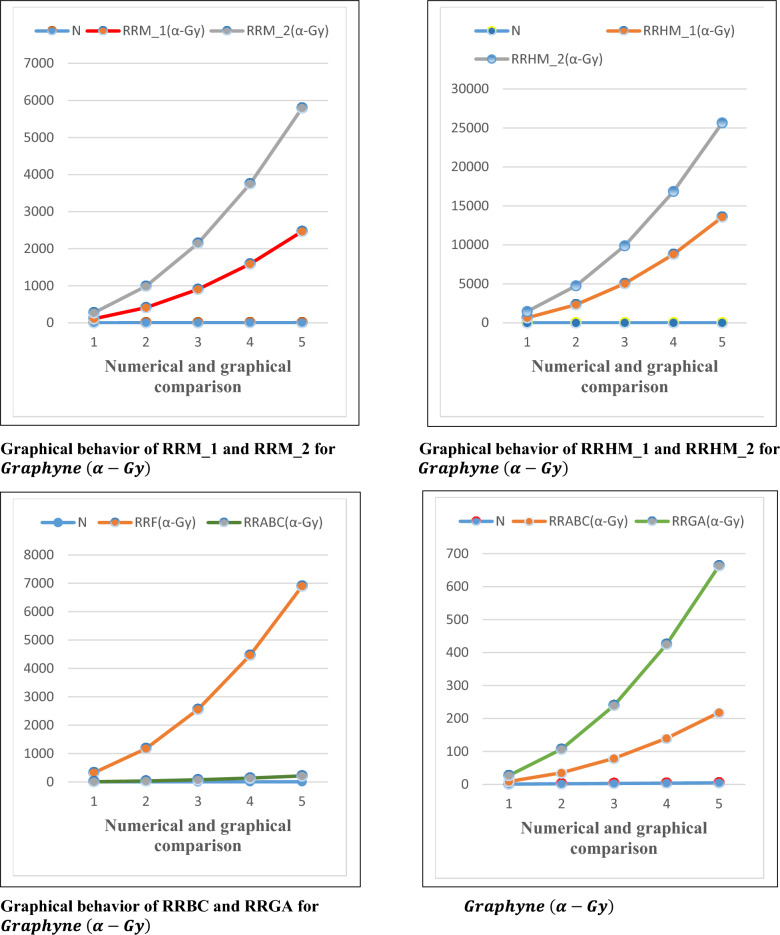
Comparison graphs for $Graphyne (\alpha -Gy)$.

**Figure 4:**
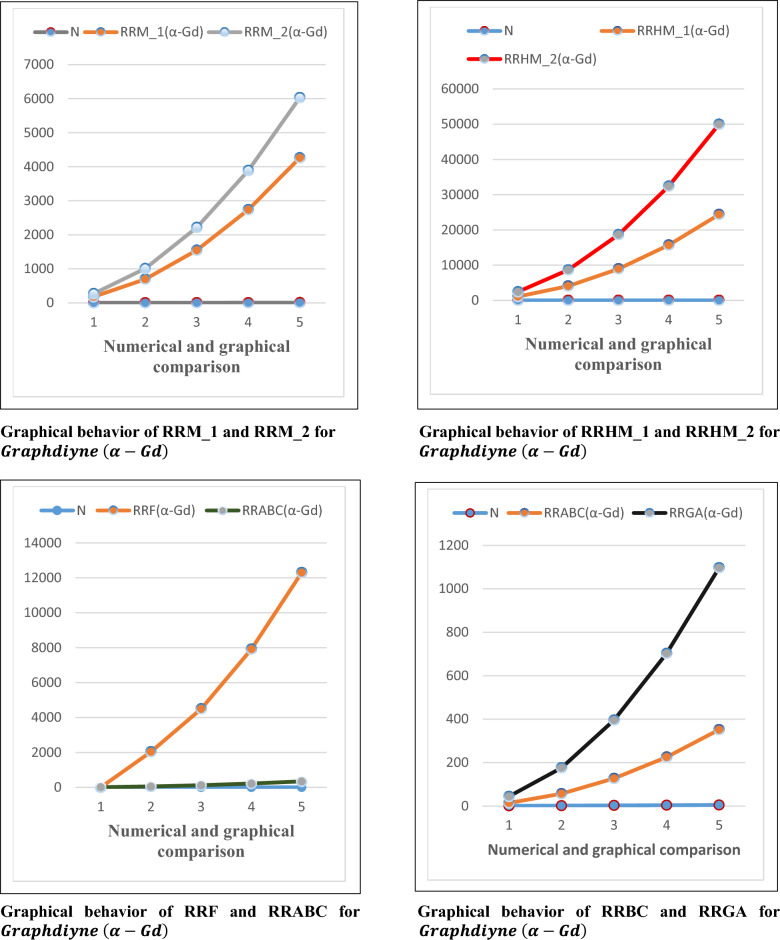
Comparison graphs for $Graphdiyne (\alpha -Gd)$.

In this article, we have calculated the frequency of reduced reverse degree-based edge partitions corresponding to $(\alpha -Gy)$ and $(\alpha -Gd)$, two different graphyne and graphdiyne atomic structures. Using these edge partitions, we have determined reduced reverse degree-based topological indices, including reduced reverse Zagreb indices, reduced reverse hyper-Zagreb indices, reduced reverse forgotten index, reduced reverse Geometric-arithmetic index (GA), and reduced reverse atom bond connectivity index (ABC), for $(\alpha -Gy)$ and $(\alpha -Gd)$**,** respectively. Finally, we have compared our obtained results in Figs. 3 and 4.

In future studies, we plan to apply these descriptors to different metal–organic framework advancements and observe the physical–chemical characteristics of various physical formations, such as silicone structures, hexagonal chains, polymers, sugars, and fullerenes. Additionally, exploring other potential applications of reduced reverse degree-based TIs in different scientific fields can be a promising avenue for future research.

## Data Availability

All data generated or analysed during this study are included in this published article.
